# Distribution of Disease

**Published:** 1889-11-23

**Authors:** 


### The Practitioner's Outlook and Retrospect.

*[The Editor will be glad to receive offers of co-operation and contributions from members of the profession. There is no doubt that much valuable material full of interest and instruction is at present lost to science. This is largely so in the case of (1) the younger and less-known members of the hospital staff, (2) those connected with cottage hospitals, and (3) the great body of medical practitioners not attached to any institution. The co-operation of all is cordially invited to enable the Editor to make* THE HOSPITAL *of the utmost possible use and value to the profession. Specialists willing to review books on their special subjects are invited to communicate this fact at once. All letters should be addressed to* THE EDITOR, THE LODGE, PORCHESTER SQUARE, LONDON, W.]

### MEDICINE.

### DISTRIBUTION OF DISEASE.

"The Geographical Distribution of Diseases and their Relation to Physical Phenomena" is the title of a most interesting article by Dr. R. W. Felkin, Lecturer on Diseases and Climatology, Edinburgh Medical School, in the *Proceedings* of the Royal Society of Edinburgh, session 1888-89. The article is amply illustrated by coloured maps, showing the distribution of various diseases throughout the world. The first map and letterpress treats of the distribution of malaria. In Europe malaria prevails most on the shores of the Black Sea and on the southern coasts of Spain. In Asia, the coasts of the Persian Gulf, especially the Indian and China coasts, and in Siam. In Africa, the Guinea coast, the eastern coasts, and Madagascar ar most malarious. In America malaria is most prevalent on the coasts of the Gulf of Mexico, on the Peruvian coasts, in Guiana, and in the Argentine Republic. The northern part of Australia is also overshadowed by the malarious colour, but the southern part is quite free. The author evidently believes in the established views regarding malaria—viz., that it is "an earth-born poison generated in the soil, the energies of which are not expended in the growth and sustenance of healthy vegetables." By almost universal consent this poison is regarded as the cause of all the types of intermittent and remittent fever, and of the degeneration of the blood and tissues arising from long residence in places where the poison is generated. There have not, however, been wanting those who question this conclusion. For instance, Ziemssen, after considering the usual views of malaria, states, "The necessary conclusion from all this is, that the telluric influences referred to are not sufficient to account for malaria." Dobell said, "We speak of it in a glib style as malaria, but in reality we know nothing about it." Surgeon-General Gordon, C.B., remarked, "If my critic will be good enough to define malaria and malarious influences as distinct from climatic and endemic influences, he will confer an important benefit on future investigators." Dr. Oldham, of the Indian Service, wrote a book, "What is Malaria?" in which he denies its existence. And Dr. Macnamara asked the pertinent question, "Is that which we called malaria the sum of the operations of the various conditions of climate and place by which we are surrounded." Since, however, these authors wrote, the Italian observers, Crudeli, Klebs, Cuboni, Marchiafava, assert they have found the *bacillus malariæ*. But malarious diseases prevail everywhere, or at least on all kinds of surfaces, and it is scarcely reasonable to presume that alluvial soils, ferruginous earths, granite and limestone rocks, marshes, dry sand, and clay, all produce the same variety of vegetable organism as described by the Italians, and, in fact, the *bacillus malariæ* has not been discovered everywhere where so-called malarious diseases prevail.

Dr. Felkin's second map relates to dengue. India, Arabia, the United States, the West India Islands, and the shores of the Carribean Sea are the principal habitats of dengue. It therefore corresponds with the belt of maximum heat throughout the globe. We are not aware that it has ever been seen in Great Britain, but it is common enough in our great Eastern Empire. It is defined as an eruptive fever, characterised by severe pain in the head, muscles, and joints, prone to shift suddenly, with catarrhal symptoms, sore throat, and swelling of the sub-maxillary glands. It therefore bears a resemblance to various other maladies, or perhaps it should be rather said it seems a compound of various other maladies, as measles, rheumatism, influenza, and common catarrh. Sometimes the rash very much resembles measles, at other times it is more like scarlet fever. It is usually very contagious, but instances are known of many persons passing through a dengue-infected country and not taking the disease.

A third map relates to cholera, and Eastern India is highly coloured as its home. There are, however, ample reasons for the assertion that although cholera is ordinarily more virulent and more general in that part of Hindustan, which Dr. Felkin vividly colours, it may yet prevail as virulently in any part of the world. Also that every epidemic of cholera is not originated and spread from that much maligned tract of our Indian Empire. Australia, we see, is coloured as one of the regions where cholera has never occurred, but recently cases have happened in that part of the world.

Oriental boil is the subject of the next map, and, of course, this is confined to Oriental countries. The author does not include Aden in the localities where Oriental boil prevails, and this is a mistake. Much discussion has arisen regarding the cause of this Oriental boil, but the balance of facts seems to warrant it being regarded as of scorbutic origin. Yellow fever is shown on the same map under a different colour. This only prevails on the west coasts of Africa and on the coasts of South America, and of the Gulf of Mexico. The author does not regard yellow fever as malarial, for he writes, "Those conditions of soil necessary for the production of malarial poison exert no influence in producing yellow fever." Yet many authorities think yellow fever malarious. The poison, it is said, clings to the ground, and its diffusion may be barred by streams, walls, and, as some assert, by much-travelled thoroughfares. So it has been said of malaria. Dr. Felkin informs us that preventive inoculations are now being largely practised against yellow fever with very great success. Another plate refers to dysentery. On this plate India again is coloured most deeply, but Brazil, the Southern States of America, and Central Africa show little less depth of colour. New Zealand is also coloured highly, but Australia is almost free. Curiously enough, Iceland is coloured for what the author calls tropical dysentery. Dr. Felkin believes that endemic dysentery is due to some microbe. But various facts prove that the disease is influenced by climate, and that heat is a necessary factor in its production. Some authorities separate dysentery into two distinct maladies—viz., contagious and non-contagious—but really there is no such distinction, and if by contagion is meant a large number of persons being attacked, malarious fever has quite as good claim to be regarded contagious as dysentery. The map on leprosy shows what has been before advanced in this journal, that the intensity of leprosy corresponds with the belt of maximum heat of the globe. There are, however, certain parts of the world not so highly coloured in which leprosy exists. These are principally New Zealand, the Cape, Iceland, Nova Scotia,

Norway, the northern parts of the Chinese Empire, and Japan. It may be doubted, however, whether the northern parts of the Chinese Empire are rightly coloured, as the Chinese regard a visit to the North as a cure for leprosy. Another plate is devoted to yaws, which chiefly prevails among Negroes, Malayians, and Polynesians. Yaws consists of an eruption of yellowish or reddish-yellow tubercles, which gradually develop into a moist exuding fungus, without constitutional symptoms. It is contagious by actual contact. Other colours relate to the fungus foot disease of India, which has not yet been found elsewhere; to elephantiasis arabum, which appears mostly to prevail on the coast of Arabia, but is common in India and Brazil; to guinea worm, which has its habitat chiefly in India, Arabia, and Africa, although it has been found in some parts of Southern Europe; and to scurvy, which we believe prevails much more extensively throughout the globe than painted by the author. Dr. Felkin's maps and letterpress are most interesting, and demand more attention and space than we are able to afford.

